# Supplementary material for: A Multi-Level Strategy Based on Metabolic and Molecular Genetic Approaches for the Characterization of Different Coptis Medicines Using HPLC-UV and RAD-seq Techniques
Source: Molecules. 2018 Nov 27;23(12):3090. doi: 10.3390/molecules23123090 (PMC6321400; doi:10.3390/molecules23123090)
Supplement: Supplementary file 1 [file molecules-23-03090-s001.pdf]

Table S1. Materials of four *Coptis* plants used in this study.

	Quantity	Producing area
C. omeiensis	10	Heishan, Hongya, Sichuan, China
C. teeta	10	Pihe, Fugong, Yunnan, China
C. chinensis	10	Heishan, Hongya, Sichuan, China
C. deltoidea	10	Heishan, Hongya, Sichuan, China

Table S2. The validation of HPLC-UV method.

Standard	Precision	Stability	Repeatability	Recovery (%)	RSD (%)
	RSD (%) (n=5)				n=3
Berberine	0.64	0.97	0.51	98.11	0.92
Palmatine	0.63	0.52	0.65	97.52	0.88
Jatrorrhizine	0.63	0.26	0.69	101.59	1.51
Coptisine	0.75	0.29	0.76	103.35	0.47
Columbamine	0.79	0.39	0.94	102.31	1.13
Epiberberine	0.51	0.84	0.79	101.12	2.16
Magnoflorine	0.37	0.46	0.78	102.38	1.12
Groenlandicine	0.95	1.09	1.95	100.65	0.69

Table S3. Statistical summary of MIDs, raw reads, filtered reads of each *Coptis* plants from RAD-seq

Species	MID	Raw reads			Clean reads		
		Raw reads count	Raw data (bp)	GC (%)	Clean reads count	Clean data (bp)	GC (%)
C. omeiensis	TCCTTGCA	10872358	1630853700	39.13	10655424	1598180450	39.08
C. teeta	ACGTACCA	11100218	1665032700	39.41	10874838	1631074106	39.36
C. chinensis	GACTGAGA	11795008	1769251200	39.41	11548500	1732152482	39.35
C. deltoidea	ATATCCGG	10979432	1646914800	38.26	10793194	1618899904	38.22

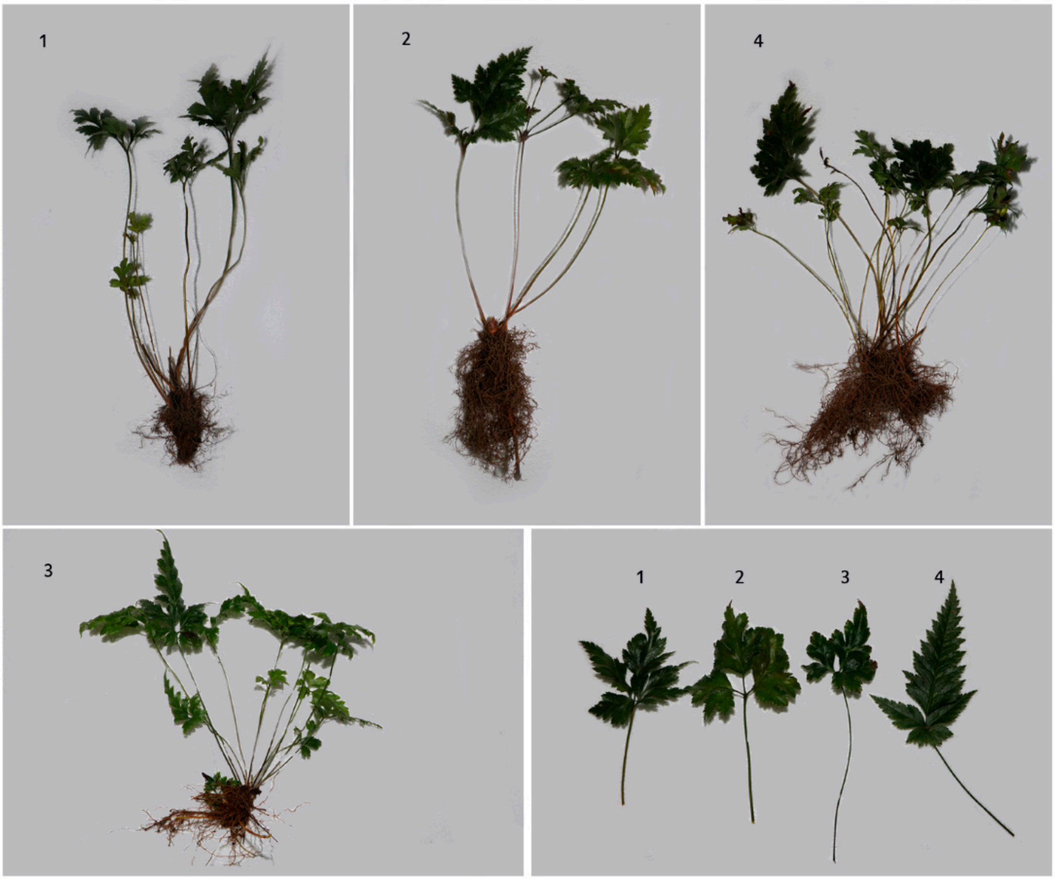

Figure S1. Plant morphology and leaf tissues of four *Coptis* plants (1: *C. chinensis*; 2: *C. deltoidea*; 3: *C. teeta*; 4: *C. omeiensis*).

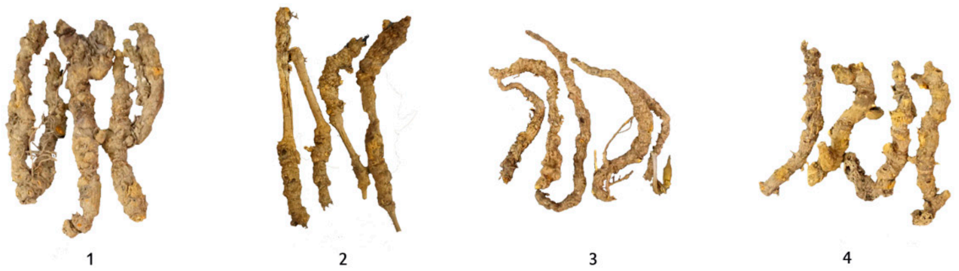

Figure S2. Rhizomes of different *Coptis* plants (1: *C. chinensis*; 2: *C. deltoidea*; 3: *C. teeta*; 4: *C. omeiensis*).

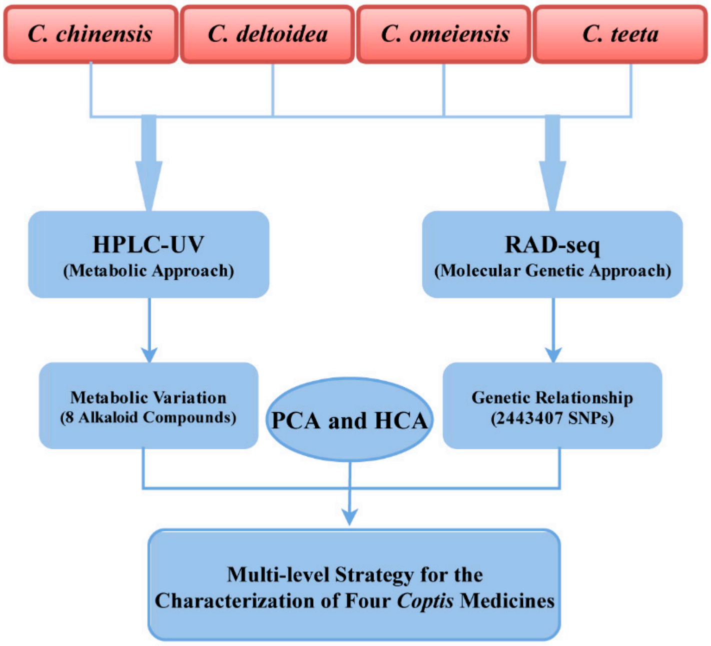

Figure S3. Data analysis flow chart of this study.

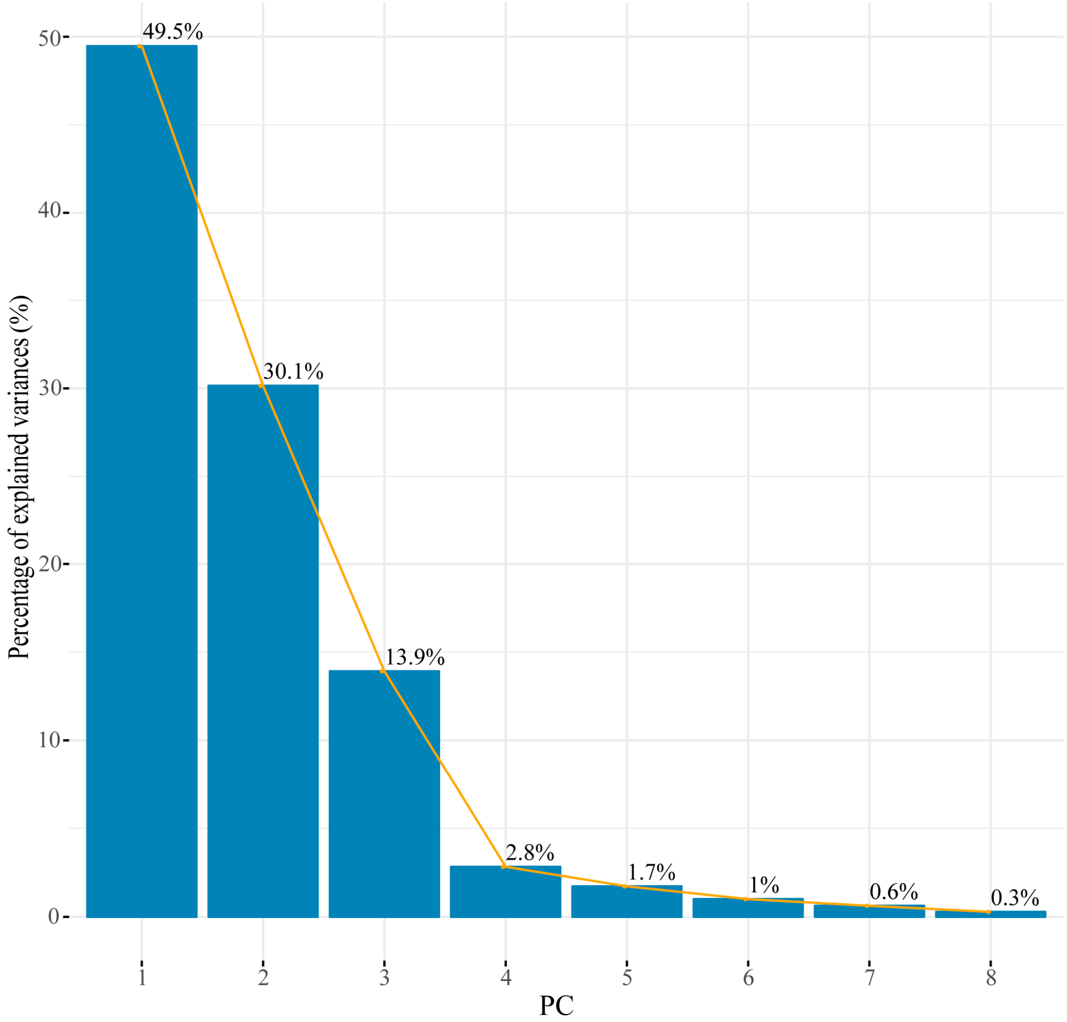

Figure S4. Principal components explaining variances used in PCA according to quantitative determination of alkaloid compounds.

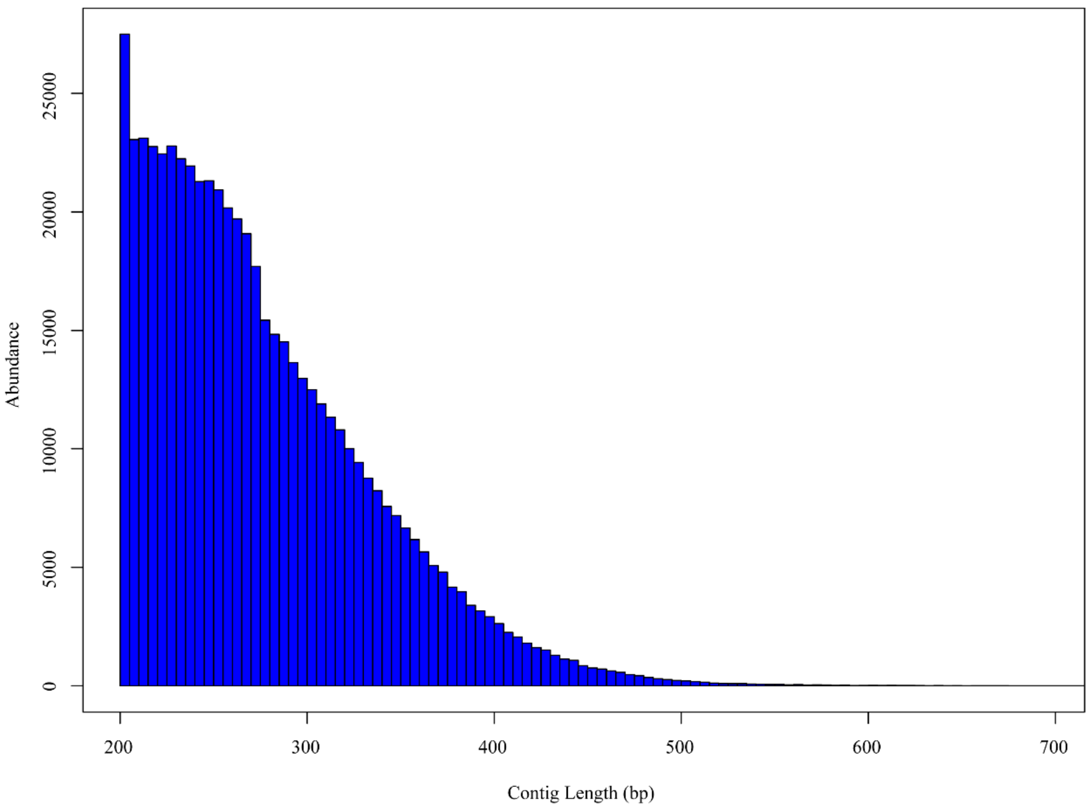

Figure S5. Length distribution of RAD-seq contigs.
